# Cetirizine and Dexamethasone in Sepsis: Insights into Maresin-1 Signaling and Cytokine Regulation

**DOI:** 10.3390/jcm15010198

**Published:** 2025-12-26

**Authors:** Yalcin Aydin, Mehmet Kazim Borku, Kader Ugur, Yesari Eroksuz, Elif Emre, Canan Akdeniz Incili, İbrahim Sahin, İlknur Zeynep Acarturk, Suleyman Aydin, Do-Youn Lee

**Affiliations:** 1 Department of Internal Medicine, Faculty of Veterinary Medicine, Ankara University, 0600 Ankara, Türkiyekborku@ankara.edu.tr (M.K.B.); 2 Department of Internal Medicine (Endocrine and Metabolism Diseases), Medical School, Firat University, 23119 Elazig, Türkiye; kaderaksoy06@hotmail.com; 3 Department of Pathology, Faculty of Veterinary Medicine, Firat University, 23119 Elazig, Türkiye; yeroksuz@firat.edu.tr (Y.E.); caincili@firat.edu.tr (C.A.I.); 4 Department of Anatomy, Medical School, Firat University, 23119 Elazig, Türkiye; elifkaplan1.1@gmail.com; 5 Department of Medical Biochemistry and Clinical Biochemistry (Firat Hormones Research Group), Medical School, Firat University, 23119 Elazig, Türkiye; isahin.1@hotmail.com; 6 Department of Medical Biology, Medical School, Erzincan Binali Yildirim University, 24100 Erzincan, Türkiye; 7 Department of İnternal Medicine, Samsun Gazi State Hospital, 55070 Samsun, Türkiye; izkilic@hotmail.com; 8 College of General Education, Kookmin University, Seoul 02707, Republic of Korea

**Keywords:** sepsis, dexamethasone, cetirizine, Maresin-1, interleukins

## Abstract

**Background/Objectives**: Sepsis remains one of the leading causes of mortality, yet its etiopathogenesis is still not fully understood. This study aimed to investigate the effects of cetirizine and dexamethasone (alone and in combination) on serum levels of Maresin-1 (MaR-1), TNF-α, IFN-γ, IL-1, IL-2, IL-6, IL-8, and IL-10 in a rat model of sepsis induced by the cecal ligation and puncture (CLP) method. **Methods**: Male Sprague Dawley rats aged 8–10 weeks were used and randomly divided into 7 groups, each containing 7 rats: Group 1 (Control), Group 2 (Sham), Group 3 (Sepsis), Group 4 (Sepsis + Saline), Group 5 (Sepsis + Cetirizine), Group 6 (Sepsis + Dexamethasone), and Group 7 (Sepsis + Cetirizine + Dexamethasone). Sepsis was induced via CLP in all groups except Control and Sham. **Results:** In the sepsis groups (G3–G7), neutrophil and white blood cell counts increased while lymphocyte counts decreased (*p* < 0.05). In groups treated with cetirizine and/or dexamethasone (G5–G7), a significant decrease in neutrophils and an increase in lymphocytes were observed. MaR-1 levels significantly decreased (*p* < 0.05) in all sepsis-induced groups compared to controls, while interleukin levels significantly increased. Cetirizine and dexamethasone supplementation significantly increased MaR-1 levels and decreased interleukin levels (*p* < 0.05). The combined treatment was more effective. **Conclusions:** This study is the first to highlight the potential of MaR-1 as a critical biomarker in sepsis diagnosis and monitoring, and cetirizine and dexamethasone, especially in combination, may represent a promising therapeutic option in sepsis management.

## 1. Introduction

Sepsis is a systemic and exaggerated inflammatory response of the body, typically triggered by infections caused by various pathogens such as bacteria [1,2]. Toxins generated in the host during the progression of sepsis activate macrophages, leading to the release of various inflammatory cytokines such as IL-1, IL-6, IL-8, and tumor necrosis factor-alpha (TNF-α), mediated through MAPK and NF-κB pathways [3]. These exotoxins have been reported to increase the production of pro-inflammatory mediators including IL-2 and IFN-γ [4]. However, excessive cytokine production may lead to tissue damage, hemodynamic changes, organ failure, and eventually death [5]. In sepsis, oxidative stress, neutrophils (NEU), and macrophages play critical roles [6].

Maresin-1 (MaR-1), derived from docosahexaenoic acid (DHA) and synthesized by monocyte-macrophage cells, is a molecule known to exert effects on cytokines by inhibiting inflammatory responses [7,8]. MaR-1 levels are reduced during inflammation [8], suggesting its potential as a negative acute-phase reactant. Thus, MaR-1 plays a role in resolving acute inflammation and protecting organs from the harmful effects of inflammation [9]. Furthermore, it contributes to clearing inflammatory infiltrates, preserving tissue integrity, and restoring tissue function [10].

A clear link exists between inflammation and cytokines (interleukins) in sepsis [3,4]. Therefore, agents capable of regulating cytokine production and attenuating or eliminating inflammation are of great interest in sepsis treatment. Antihistamines such as cetirizine [11] and corticosteroids like dexamethasone [12] are agents known for their anti-inflammatory properties. Antihistamines can activate glucocorticoid receptor genes involved in inflammation, thereby enhancing the anti-inflammatory effects of dexamethasone [13]. In wild-type mice, levels of monocyte chemoattractant protein (MCP)-1, IL-1, IL-6, and TNF-α—elevated after sepsis induction—have been shown to decrease with cetirizine treatment [14]. Moreover, systemic corticosteroid use shortens the duration of septic shock [15], and in animal models, dexamethasone has been shown to reduce inflammation, cerebral edema, intracranial pressure, and brain injury [16]. Dexamethasone administered prior to endotoxin challenge also reduces TNF-α production [17].

Previous research has investigated the anti-inflammatory role of MaR-1 (for example, in sepsis-induced acute kidney injury), but not specifically as a serum diagnostic biomarker [18]. Corticosteroids reverse organ failure and improve the partial pressure of oxygen/fraction of inspired oxygen (PO2/FiO2) ratio in septic shock patients [19]. In addition, the combined use of antihistamines in sepsis has also been reported in earlier studies (e.g., improvement of urine output in septic shock) [20,21].

To the best of our knowledge, however, cetirizine and dexamethasone medication combinations both alone and in combination have not yet been studied. Therefore, this study aimed to investigate the effects of cetirizine and dexamethasone (alone and in combination) on serum levels of MaR-1, TNF-α, IFN-γ, IL-1, IL-2, IL-6, IL-8, and IL-10 in a rat model of sepsis induced by CLP method.

## 2. Materials and Methods

This study was conducted at the Fırat University Animal Research Center with the approval of the for Fırat University Local Ethics Committee Animal Experiments (FÜHADYEK), under the decision numbered 2021/01 dated 6 January 2021. A total of 49 male Sprague-Dawley rats, aged 8–10 weeks and weighing between 281–312 g, were used. The animals were randomly assigned into seven groups, each consisting of seven rats: one control group and six experimental groups.

Before the experiment, the rats were housed in the FÜDAM laboratory under controlled conditions (22–25 °C, 12 h light/dark cycle). The bedding was changed daily, and they were provided with food and water and libitum. The rat feed was prepared by Elazığ Yem Sanayi A.Ş., and its content was as previously described [22].

Sepsis was induced using the cecal ligation and puncture (CLP, punctured the cecum using a 19-gauge needle to take a single pass through the cecum) model, as described by Wichterman et al. [23]. After CLP, fecal content discharge was observed. The groups were designed as follows: **Group 1 (Control Group, n: 7):** No surgical or pharmacological intervention was performed. **Group 2 (Sham Group, n: 7):** The abdomen was opened, and the intestines were exteriorized and then repositioned without any intervention; no treatment was given. **Group 3 (Sepsis Group, n: 7):** The abdomen was opened, the cecum was ligated and punctured, and fecal content was extruded into the peritoneal cavity to induce sepsis. The intestines were repositioned, and the abdomen was closed. **Group 4 (Sepsis + Saline Group, n: 7):** Sepsis was induced as in Group 3. One-hour post-CLP, the rats received 1 mL/kg of saline intraperitoneally (i.p.). **Group 5 (Sepsis + Cetirizine Group, n: 7):** Sepsis was induced as in Group 3. One hour later, rats received 1 mL/kg of saline (i.p.) and 1 mg/kg of oral cetirizine. **Group 6 (Sepsis + Dexamethasone Group, n: 7):** Sepsis was induced as in Group 3. One-hour post-CLP, 1 mL/kg of saline (i.p.) and 1 mg/kg of dexamethasone (i.p.) were administered. **Group 7 (Sepsis + Dexamethasone + Cetirizine Group, n: 7):** Sepsis was induced as in Group 3. One-hour post-CLP, rats received 1 mL/kg of saline (i.p.), 1 mg/kg of dexamethasone (i.p.), and 1 mg/kg of oral cetirizine. The cetirizine [24] and dexamethasone doses [25] used in these topics were based on previous studies.

### 2.1. Surgical Procedures, Sample Collection and Preparation

**Figure 1:**
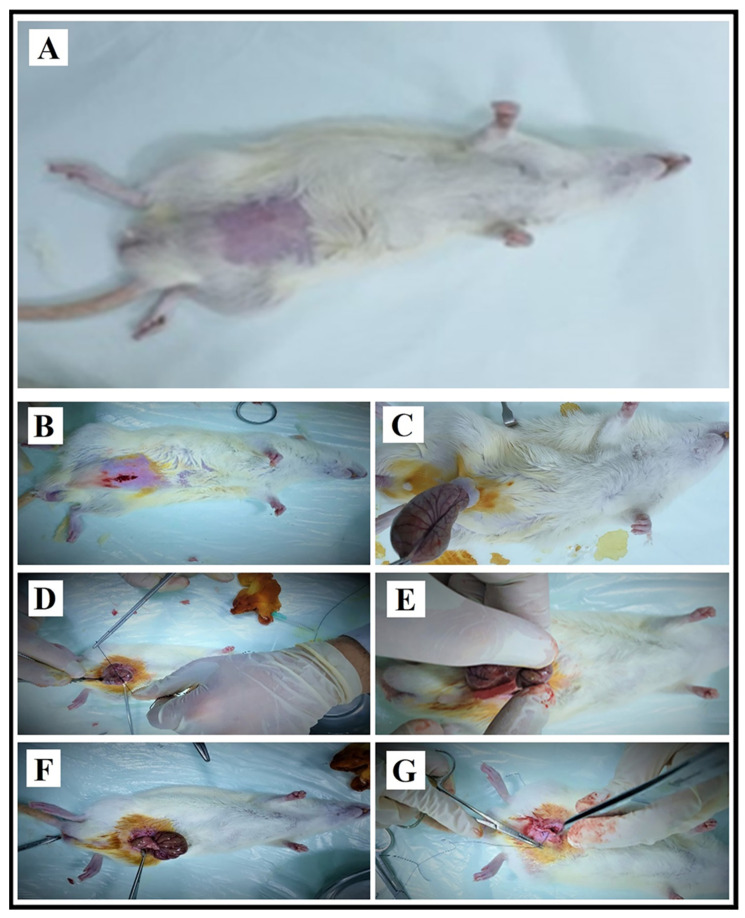
Stages of surgical interventions applied to induce sepsis in the experimental group rats. (**A**): Shaving the abdomen to prevent contamination before surgical intervention. (**B**): Opening the median line in the prepared operation area and accessing the abdomen. (**C**): Removal of the cecum and intestines from the abdomen. (**D**): Ligation of the removed cecum. (**E**): Puncture of the ligated cecum and removal of stool. (**F**): Infecting the peritoneum with stool removed from the cecum. (**G**): After the cecum and intestines are placed in the abdomen, 2 cc of physiological serum is administered to the abdomen and the peritoneum, abdominal muscles and skin are sutured.

Before the CLP procedure (A schematic representation of the surgical steps used for sepsis induction is provided in Figure 1), xylazine (10–15 mg/kg) and ketamine (50–80 mg/kg) were administered intramuscularly. Additional anesthetic doses were administered to maintain normal breathing during the experiment; the disappearance of the righting reflex was considered the criterion for adequacy of anesthesia. Surgery was performed after anesthesia was achieved.

During the surgical procedure, a midline incision was made in the animals’ abdomen to provide access to the intra-abdominal organs. In the non-sepsis-induced groups, the cecum was removed; placed back into the abdominal cavity without any surgical intervention, and the abdomen was closed. In the sepsis-induced groups, the cecum was removed; its blind end was ligated with 3-0 silk suture. The ligated area was then punctured to facilitate fecal drainage. The fecal output was then brought into contact with the abdominal cavity to create a sepsis model. Before the cecum was repositioned into the abdomen, 1 mL of saline was administered intraperitoneally to the respective groups. Following completion of all procedures, the intestines were placed in their anatomical positions, and the abdomen was closed with two or three surgical wound clips. All surgical procedures were performed under sterile conditions and in accordance with standard protocols (Wichterman et al.) [23]. At the 16th hour, the end of the experimental process, the experimental animals were sacrificed using capital decapitation without the administration of any anesthetic. Additionally, animal tissues and blood were obtained using recently updated [26] and previously published protocols [27]. Tissues were placed in 10% formaldehyde and sent to the laboratory. Blood samples were centrifuged at 4000 rpm, and the resulting sera were stored in Eppendorf tubes until −80 °C.

### 2.2. Histopathology

For histopathological analysis, tissue samples were collected post-mortem from the liver, kidney, spleen, eye, and both the small and large intestines. All samples were fixed in 10% neutral buffered formalin, routinely processed, and embedded in paraffin blocks. Serial sections of 5–6 µm thickness were obtained using a rotary microtome and stained with hematoxylin and eosin (H&E).

Histopathological evaluation of hepatic inflammation and injury was performed using a semi-quantitative scoring system, in which portal and parenchymal inflammatory infiltration, venous dilatation, sinusoidal congestion, and hepatocellular necrosis were assessed. Each parameter was graded on a three-point scale, defined as 1 (absent or mild), 2 (moderate), and 3 (severe).

### 2.3. Biochemical Analyses

In this study, serum levels of MaR-1, TNF-α, IFN-γ, IL-1, IL-2, IL-6, IL-8, and IL-10 were determined using the ELISA method at the Biochemistry Laboratory of the Faculty of Medicine, Fırat University. The ELISA kits are used, and their specifications are as follows: MaR-1: Cat. no. 201127349, Lot no. 202104202110 (Cayman, Ann Arbor, MI, USA). TNF-α: DZE SRB-T-82883, Lot no. 202102-2021108 (Sunred Biological Technology Co., Ltd., Shanghai, China). IFN-γ: (Sunred Biological Technology Co., Ltd., Shanghai, China). IL-1: Cat. no. E0107 Ra, Lot no. 202202002 (BT Lab, Bioassay Technology Laboratory, Shanghai, China). IL-2: Cat. no. E0123 Ra, Lot no. 202202002 (BT Lab, Bioassay Technology Laboratory, Shanghai, China). IL-6: Cat. no. DEE SRB-T-83168, Lot no. 202107-202201 (Sunred Biological Technology Co., Ltd., Shanghai, China). IL-8: Cat. no. DZE 20110138, Lot no. 2021107-202201 (Sunred Biological Technology Co., Ltd., Shanghai, China). IL-10: Cat. no. DZE SRB-T-83478, Lot no. 202107-202201 (Sunred Biological Technology Co., Ltd., Shanghai, China).

All ELISA procedures were conducted according to the manufacturers’ protocols. The coefficients of variation (CV) for intra-assay ranged from 4.6% to 10%, and for inter-assay from 10% to 15%. The analytical validity of the tests—including specificity, sensitivity, linearity, intra-assay, and inter-assay variability—was confirmed [26,27].

For washing ELISA plates, the Bio-Tek ELX50 device was used, and absorbance readings were taken using the ChroMate Microplate Reader P4300. Additionally, white blood cell (WBC), platelet (PLT), neutrophil (NEU), lymphocyte (LYM), and monocyte (MO) counts were measured from whole blood samples at the Microbiology Laboratory of Malatya Turgut Özal University Training and Research Hospital.

### 2.4. Statistical Analyses

Statistical analyses were performed using the SPSS 22.0 software package. Normality levels of distributions tested by Shapiro–Wilk test, while the Kruskal–Wallis H test (Dunn’s post hoc) was used to compare parameters across multiple groups. Potential correlations between analyzed variables were assessed using the Spearman correlation test. All statistical results were expressed as mean ± standard deviation, rounded to two decimal places. A *p*-value of <0.05 was considered statistically significant.

## 3. Results

There was no statistically significant difference (*p* > 0.05) in body weights between the control [(G1: 281.64 g ± 8.41 g)] and experimental groups [(G2: 281.83 g ± 6.04 g); (G3: 287.85 g ± 8.93 g); (G4: 298.85 g ± 24.70 g); (G5: 299.50 g ± 23.89 g); (G6: 310.50 g ± 40.10 g); (G7: 312.42 g ± 45.32 g)]. All rats were physically healthy prior to the experiment. At the 4th hour after the beginning of the experiment, rats were still recovering from anesthesia and showed signs of lethargy. By the 16th hour, rats in the control (G1) and sham (G2) groups appeared healthy, whereas rats in the sepsis groups displayed generalized weakness, decreased responsiveness to external stimuli, and reduced mobility.

No significant difference (*p* > 0.05) was found in pre-experiment body temperature values between the control and experimental groups. The body temperature recorded just before the start of the experiment was taken as the baseline for all experimental comparisons. A statistically significant drop in temperature was observed at the 4th hour post-induction (*p* < 0.05), followed by a significant increase by the 16th hour in all groups except G1 and G2 (*p* < 0.05). Body temperature data of control and experimental groups are presented in Table 1.

### 3.1. Histopathological and Hematological Findings

**Figure 2:**
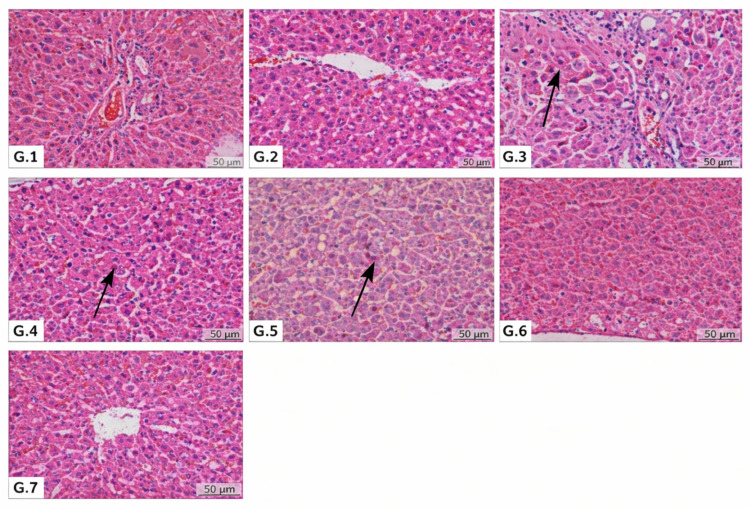
Representative histopathological images of liver tissue stained with hematoxylin and eosin (H&E) (G.1–G.7). (**G.1**) Normal hepatic architecture with radiating hepatic cords and absence of inflammatory infiltration or hepatocellular necrosis in Group 1 (H&E, ×40, scale bar = 20 µm). (**G.2**) Preserved hepatic parenchyma without hepatocellular necrosis and with moderate Kupffer cell activation (arrow) in Group 2 (H&E, ×40, scale bar = 20 µm). (**G.3**) Focal hepatocellular necrosis (arrow) accompanied by mild periportal inflammatory infiltration in Group 3 (H&E, ×40, scale bar = 20 µm). (**G.4**) Focal hepatocellular necrosis (arrow) and Kupffer cell activation in Group 4 (sepsis + cetirizine) (H&E, ×40, scale bar = 20 µm). (**G.5**) Reduced hepatocellular necrosis (arrow) and Kupffer cell activation, with improved preservation of hepatic architecture, in Group 5 (H&E, ×40, scale bar = 20 µm). (**G.6**) Near-normal hepatic histology with no evident necrosis or Kupffer cell activation in Group 6 (H&E, ×40, scale bar = 20 µm). (**G.7**) Near-normal hepatic architecture without necrosis or inflammatory changes in Group 7.

Histopathological alterations were exclusively confined to the liver, while no remarkable microscopic lesions were detected in the kidney, heart muscle, spleen, intestines, or eye. Hepatic lesions were characterized by focal hepatocellular degeneration and necrosis, periportal lymphohistiocytic infiltration, Kupffer cell activation, and congestion of venous and sinusoidal compartments. These changes were absent or minimal in the control (G1) and sham-operated (G2) groups (Figure 2).

Periportal inflammatory infiltrates were composed predominantly of lymphocytes and macrophages, with occasional neutrophils. Necrotic hepatocytes were observed singly or in small clusters, showed hypereosinophilic cytoplasm and pyknotic nuclei, and lacked a distinct lobular or zonal distribution (Figure 2). Notably, focal lymphohistiocytic infiltrates interpreted as post-necrotic inflammatory foci were observed exclusively in the sepsis group (G3).

Quantitative histopathological scoring confirmed that Group 3 (sepsis) exhibited the most severe hepatic injury across all evaluated parameters, including inflammation (2.17 ± 0.41), hepatocellular necrosis (2.83 ± 0.75), vascular congestion (2.50 ± 0.84), and Kupffer cell activation (3.00 ± 0.00). Among the treatment groups, Group 7 (sepsis + dexamethasone + cetirizine) demonstrated the greatest reduction in hepatocellular necrosis (2.20 ± 0.84).

Vascular congestion, which was markedly pronounced in Group 3, was significantly reduced in Group 6 (dexamethasone alone; 1.67 ± 0.82) and Group 7 (1.80 ± 0.84), indicating a beneficial effect of dexamethasone on sepsis-associated hepatic microcirculatory disturbances. Kupffer cell activation remained elevated in all septic groups; however, partial attenuation was observed in Group 5 (cetirizine) and Group 7 (Table 2).

Collectively, these findings indicate that both cetirizine and dexamethasone provide partial histopathological protection against sepsis-induced hepatic injury. Importantly, their combined administration (Group 7) resulted in the most consistent improvement across all histological parameters, supporting a potential additive or synergistic therapeutic effect in the mitigation of sepsis-related liver damage.

In hematological evaluations (at both the 4th and 16th hours), there were no statistically significant differences (*p* > 0.05) in WBC, PLT, NEU, or MO levels between the sham (G2) and control (G1) groups (Table 3). In contrast, statistically significant increases (*p* < 0.01) in these parameters were observed in the other experimental groups (G3–G7). Although an increase in lymphocyte (LYM) counts was observed in groups treated with cetirizine and/or dexamethasone (G5, G6, G7), this increase was not statistically significant (*p* > 0.05) except for 16th hour G7 group, and 4th hour G5 group.

At the 16th hour, compared to the control (G1) and sham (G2) groups, LYM levels decreased while NEU levels increased in all sepsis groups. Notably, treatment with cetirizine (G5), dexamethasone (G6), or their combination (G7) led to an increase in LYM and a decrease in NEU levels (Table 3).

### 3.2. Serum MaR-1, TNF-α, IL-1, and IFN-γ Levels

**Figure 3:**
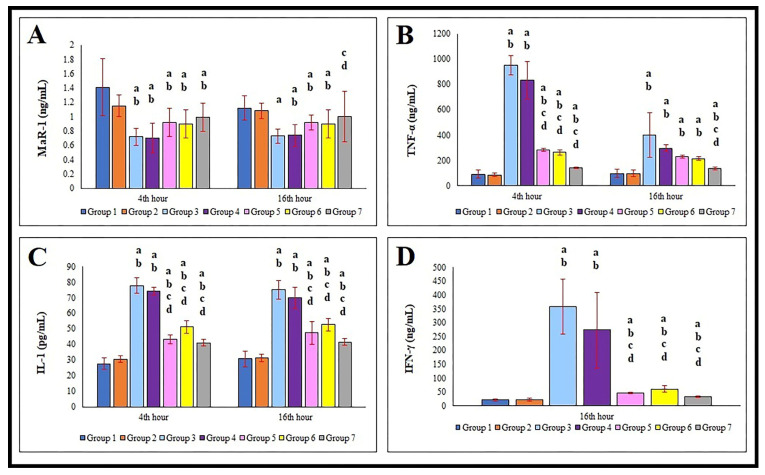
Comparison of MaR-1 (**A**), TNF-α (**B**), IL-1 (**C**), and IFN-γ (**D**) in serum of the control and experimental groups. ^a^: comparison vs. G1; ^b^: comparison vs. G2; ^c^: comparison vs. G3; ^d^: comparison vs. G4. Statistical comparisons between groups were evaluated using the Kruskal–Wallis H test (Dunn’s post hoc test). The *p* value of all comparisons is less than 0.05. MaR-1: Maresin-1. TNF-α: Tumor Necrosis Factor-alpha. IL: Interleukin. IFN-γ: Interferon-gamma. G: Group. Group 1: Control. Group 2: Sham. Group 3: Sepsis. Group 4: Sepsis + Saline. Group 5: Sepsis + Cetirizine. Group 6: Sepsis + Dexamethasone. Group 7: Sepsis + Cetirizine + Dexamethasone.

Compared to the control group (G1), serum MaR-1 levels were significantly decreased (*p* < 0.05) at both the 4th and 16th hours in all other groups (G2–G7). When the magnitude of reduction was compared between groups, the most pronounced decrease was observed in the sepsis (G3) and saline-treated sepsis (G4) groups. In contrast, the groups treated with cetirizine (G5), dexamethasone (G6), or both (G7) exhibited less reduction in MaR-1 levels. This data is presented in Figure 3A.

There was no statistically significant change in serum TNF-α levels between the control (G1) and sham (G2) groups at either time point (*p* > 0.05). However, a significant increase (*p* < 0.05) was observed in all other groups (G3–G7). The highest increases were detected in the G3 and G4 groups. The TNF-α elevation in G5, G6, and G7 were notably lower compared to G3 and G4, as shown in Figure 3B.

Regarding IL-1, the lowest serum levels were recorded in the control group (G1). A significant increase (*p* < 0.05) was observed in all other groups (G3–G7), with the highest levels again in G3 and G4. Groups treated with cetirizine (G5), dexamethasone (G6), or their combination (G7) exhibited lower IL-1 levels than the untreated sepsis groups, as shown in Figure 3C.

At the 16th hour, serum IFN-γ levels in the control (G1) and sham (G2) groups remained unchanged. However, all other groups (G3–G7) showed a highly significant increase (*p* < 0.01). The highest levels were again observed in G3 and G4. Groups G5, G6, and G7 had lower IFN-γ levels compared to G3 and G4. These results are depicted in Figure 3D. 

### 3.3. Serum IL-2, IL-6, IL-8, and IL-10 Levels

**Figure 4:**
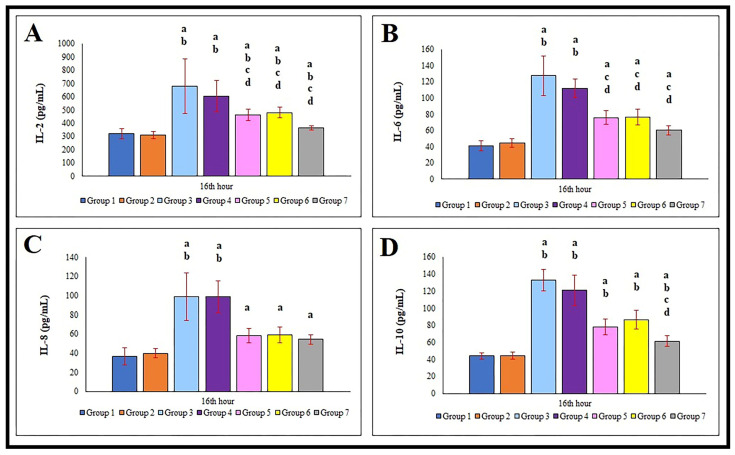
Comparison of IL-2 (**A**), IL-6 (**B**), IL-8 (**C**), and IL-10 (**D**) in serum of the control and experimental groups. IL: Interleukin. Group 1: Control. Group 2: Sham. Group 3: Sepsis. Group 4: Sepsis + Saline. Group 5: Sepsis + Cetirizine. Group 6: Sepsis + Dexamethasone. Group 7: Sepsis + Cetirizine + Dexamethasone. Comparisons and statistical significance as the same as in Figure 3 legends.

At the 16th hour, the lowest IL-2 levels were observed in the control (G1) and sham (G2) groups. Compared to these two groups, all other groups (G3–G7) exhibited a highly significant increase in IL-2 levels (*p* < 0.01). The highest IL-2 concentrations were detected in the sepsis (G3) and saline-treated (G4) groups (*p* < 0.05). Groups treated with cetirizine (G5), dexamethasone (G6), and especially their combination (G7) showed significantly lower IL-2 levels (*p* < 0.05) than G3 and G4 (Figure 4A).

At the 16th hour, all sepsis groups (G3–G7) demonstrated a highly significant increase in serum IL-6 levels compared to the control group (G1) (*p* < 0.01). The most pronounced increases occurred in G3 and G4. However, in the groups treated with cetirizine (G5), dexamethasone (G6), or both (G7), IL-6 levels were significantly lower than in G3 and G4 (Figure 4B).

Analysis of IL-8 levels revealed that the lowest levels were again found in the control group (G1). All sepsis groups (G3–G7) showed a highly significant increase in IL-8 levels compared to G1 (*p* < 0.01). The highest levels were observed in G3 and G4. In contrast, the increase was less pronounced in G5, G6, and especially G7 (Figure 4C).

At the 16th hour, there were no significant differences in IL-10 levels between the control (G1) and sham (G2) groups. However, all other groups (G3–G7) exhibited a highly significant increase in IL-10 levels (*p* < 0.01). The highest IL-10 concentrations were found in G3 and G4, while levels were lower in G5, G6, and especially G7 (Figure 4D).

No macroscopic abnormalities were observed in any of the control, sham, or experimental groups. Correlation analysis of the measured variables revealed numerous statistically significant relationships, both positive (directly proportional) and negative (inversely proportional). Overall, the number of positive correlations significantly exceeded negative ones. The details of these relationships are presented in Table 4.

## 4. Discussion

Sepsis is a life-threatening pathological process whose global incidence continues to rise [28,29,30]. Therefore, in this study, the widely used cecal ligation and puncture (CLP) model was employed to induce experimental sepsis, aiming to contribute to the understanding of its etiology [23,29,31,32].

In this study, it was observed that body temperature decreased at the 4th hour in the experimental groups. This reduction in body temperature may be attributed to anesthesia-induced immobility, which reduces oxygen consumption in the muscles and subsequently lowers energy expenditure, as previously reported [33,34,35]. Moreover, it has been reported that steroids reduce IL-1 production and thereby decrease fever in sepsis [36], suggesting that dexamethasone administration may also have contributed to the observed hypothermia.

The present histopathological findings demonstrate that CLP-induced sepsis results in significant hepatic injury, characterized by inflammation, hepatocellular necrosis, vascular congestion, and marked Kupffer cell activation, consistent with the established pathophysiology of sepsis-associated organ dysfunction [2,3]. Treatment with dexamethasone and cetirizine, particularly when administered in combination, attenuated these pathological alterations, suggesting complementary anti-inflammatory and immunomodulatory effects.

Dexamethasone likely exerts its hepatoprotective effects through suppression of pro-inflammatory cytokine release and inhibition of NF-κB signaling pathways [19]. Cetirizine, in addition to its antihistaminic properties, may modulate leukocyte activation and reduce inflammatory cell infiltration within hepatic tissue [11,13]. The superior histopathological improvement observed in the combination group supports previous evidence indicating a synergistic interaction between corticosteroids and antihistamines in inflammatory conditions [22]. Overall, these results emphasize the potential value of dual-targeted therapeutic strategies in mitigating sepsis-induced hepatic damage.

Additionally, in line with previous studies [29,37,38] this study found that at the 4th hour following surgery, LYM levels decreased, although PLT and NEU levels rose in sepsis groups, this was not statistically significant. However, administration of cetirizine and dexamethasone—either individually or in combination—led to increased LYM levels and lowered PLT and NEU levels. It is well-established that antihistamines and corticosteroids, whether used separately or together, suppress neutrophil activity and reduce inflammation [11,39]. Therefore, we propose that cetirizine and dexamethasone, as administered in this study, may have contributed to reduced NEU counts by mitigating the inflammatory response. Compared to the control and sham groups, MaR-1 levels were significantly reduced in all sepsis groups. However, the extent of this reduction was less in the groups treated with cetirizine, dexamethasone, or their combination. Cetirizine has demonstrated both anti-inflammatory and anti-allergic activity under in vitro and in vivo conditions [11]. In the present study, dexamethasone also increased MaR-1 levels. Given its potent anti-inflammatory properties [40], dexamethasone likely enhanced MaR-1 levels by reducing systemic inflammation. Furthermore, dexamethasone may protect the host from excessive immune responses in sepsis by inhibiting overactive defense mechanisms.

Dexamethasone is known to inhibit cytokine production [41,42]. Several studies have reported that MaR-1 administration improves survival rates, reduces inflammation, and lowers bacterial load in sepsis [40,43,44,45,46,47]. As a major component of Gram-negative bacterial cell walls, lipopolysaccharides (LPS) can trigger fatal inflammatory responses, leading to multiple organ failure and death. However, reduced LPS levels have been reported in mice treated with MaR-1 [43,46]. Another study showed that MaR-1 therapy mitigated renal tissue damage and improved kidney function during inflammation [47]. Collectively, these findings suggest that MaR-1 reduces systemic inflammation and mortality in sepsis and may serve as a valuable biomarker for diagnosis and follow-up.

TNF-α is the first pro-inflammatory cytokine released into circulation during sepsis [48]. In one study, TNF-α levels increased within 60–90 min following endotoxin administration in animals [49]. Another study in septic calves also showed elevated TNF-α levels [50], and an increase in neutrophil counts has been associated with elevated TNF-α in sepsis [51]. Similarly, in the present study, TNF-α levels were significantly elevated (*p* < 0.05) in all sepsis groups (G3–G7) compared to the control (G1) and sham (G2) groups. However, the elevation was notably lower in the groups treated with cetirizine and dexamethasone (G5, G6, G7).

It has been reported that MaR-1 influences TNF-α levels in sepsis and that low MaR-1 levels are associated with lower TNF-α concentrations [52]. This aligns with our findings, where groups with lower MaR-1 levels also showed reduced TNF-α levels. Furthermore, studies have shown that prednisone administration reduces serum TNF-α levels in septic patients [53], consistent with the lower TNF-α levels observed in the dexamethasone group in our study. Additionally, histamine released by mast cells is thought to inhibit TNF-α secretion, thus reducing its serum concentration [54], which may explain the lower TNF-α levels in the cetirizine-treated group. It is also known that low IL-10 levels, as seen in our study, may contribute to the reduction in TNF-α [55].

IL-1 is secreted by B-cells, keratinocytes, monocytes, mesangial cells, dendritic cells, and fibroblasts [56]. In rats with CLP-induced sepsis, elevated IL-1 levels contribute to hemodynamic shock, high fever, tissue damage, and metabolic disturbances. Additionally, increased IL-1 levels in patients with Gram-negative bacteremia have been associated with the severity of sepsis [37,45,57,58,59]. Steroids are known to reduce IL-1 production and lower fever during sepsis [36]. Consistent with this, in our study, administration of cetirizine or dexamethasone individually decreased IL-1 levels, and the combination of both agents resulted in an even greater reduction. Since antihistamines also influence glucocorticoid receptor activity associated with inflammation, they may enhance the efficacy of dexamethasone [13]. This likely explains why the group receiving both cetirizine and dexamethasone exhibited the lowest IL-1 levels.

IFN-γ is a cytokine that modulates the immune response through the regulation of phagocytic and natural killer (NK) cells [60]. It plays a key role in both infection and inflammation during sepsis. It has been observed that IFN-γ is released at low levels in the early phase of infection, and its release increases as inflammation progresses [61,62]. Studies on septic shock have demonstrated that IFN-γ contributes to the pathogenesis of sepsis, and that neutralization of IFN-γ can reduce mortality, indicating that elevated IFN-γ levels are associated with poor clinical outcomes [63,64,65,66]. In our study, IFN-γ levels were significantly elevated (*p* < 0.01) in all sepsis groups compared to controls, but treatment with cetirizine or dexamethasone reduced these levels, with the greatest reduction observed when both agents were used together. These findings support the hypothesis that cetirizine and dexamethasone can attenuate IFN-γ-mediated inflammatory responses.

IL-2 is produced by activated T-cells and CD4+ helper T-cells [67,68], and exhibits both pro- and anti-inflammatory functions [69,70]. Elevated IL-2 levels have been reported in septic shock [71]. Similarly, in our study, IL-2 levels significantly increased (*p* < 0.01) in the sepsis groups compared to controls. Treatment with cetirizine or dexamethasone individually reduced IL-2 levels, while their combined administration led to a more pronounced decrease. These results are in line with the findings of Endo et al. [71].

IL-6 is a pro-inflammatory cytokine that serves as a rapid indicator of inflammation and assists in diagnosing infection during sepsis [72,73,74]. In dogs admitted to intensive care, elevated IL-6 levels have been reported as a potential positive prognostic marker [75]. Similar increases in IL-6 have also been observed in septic calves [50]. A study using Wistar rats likewise showed that IL-6 levels rise during sepsis and suggested that this could serve as an early marker of the condition [76]. In our study, IL-6 levels were significantly elevated (*p* < 0.01) in the sepsis groups compared to controls. Cetirizine or dexamethasone alone reduced IL-6 levels, and the combination of both produced an even more notable reduction. These results support the view that IL-6 plays a critical role in the pathophysiology of sepsis and that treatment strategies should aim to reduce IL-6 concentrations [13,76,77,78,79,80].

Neutrophil activation and elevated IL-8 levels have been linked with clinical, biochemical, and inflammatory parameters in sepsis [51,81,82,83]. Increased IL-8 levels have also been documented in septic calves [50]. A meta-analysis reported that IL-8 could serve as a potential biomarker for diagnosing neonatal sepsis [84]. In burn-related sepsis, IL-8 levels have been suggested as a valid marker for infection and mortality [85]. In our study, IL-8 levels were significantly elevated (*p* < 0.01) in all sepsis groups compared to controls. Treatment with cetirizine or dexamethasone reduced IL-8 levels, and the combination of both led to an even greater decrease. These findings further highlight the importance of IL-8 in the pathophysiology of sepsis and suggest that therapeutic approaches should aim to suppress IL-8 levels [79].

Elevated IL-10 levels have been associated with clinical deterioration in patients with sepsis [86]. In CLP-induced sepsis models, IL-10 levels were found to increase [37,87]. Calzavacca et al. (2014) also observed elevated IL-10 in their sepsis study [88]. In a porcine model, IL-10 levels peaked at the 16th hour of sepsis [89]. Consistent with these findings, IL-10 levels in our study significantly increased (*p* < 0.01) in the sepsis groups compared to the control and sham groups. Treatment with cetirizine or dexamethasone individually reduced IL-10 levels, and their combination led to a further decrease. According to Oberholzer et al. [86], elevated IL-10 levels are associated with poor clinical outcomes. In line with previous reports [29,90], our study also showed inflammatory and necrotic changes in the liver in sepsis groups, which were reduced by cetirizine or dexamethasone treatment—especially when used in combination. According to a prior study, dexamethasone therapy restored the inflammation caused by cholestasis, as evidenced by histological findings and a significant decrease in many inflammatory markers (TNF-α, IL-1β, and IL-6) [91]. Additionally, antihistamine increased the overall antioxidant level, according to another study [92]. According to this present study and earlier researchers, concurrent use of these drugs (dexamethasone and antihistamine, cetirizine) may considerably reduce oxidative stress and inflammation in the event of sepsis. These results may eventually result in more efficient use of both medications in the clinic for sepsis.

As with every study, this study has some limitations. First of all, gender may influence the outcomes (e.g., hormonal differences in inflammatory responses). We suggest that future studies include both sexes. The study’s second drawback is that 16 h of follow-up were conducted following the induction of sepsis. Future research should incorporate long-term follow-up and survival analysis due to the chronic nature of sepsis. Third restriction is that in this investigation, both control (nothing provided group) and sham group (solvent of the compounds utilized) groups were present simultaneously. There was no statistical difference between the sham and control groups we therefore recommended that in future research the other researchers employ the 3R principles (Replacement, Reduction, Refinement) to avoid unnecessary animal exploitation. A last limitation of our work is that our study did not establish a link between MaR-1 changes and cetirizine/dexamethasone synergy (e.g., receptor/kinase tests). Future research should consider this. Also, NF-κB is a key transcription factor that causes inflammation and controls genes involved in innate and adaptive immune responses. Future research should consider this as well.

## 5. Conclusions

Sepsis is a complex pathological condition that requires early intervention. At the 4th hour following CLP-induced sepsis, levels of NEU, IL-1, and TNF-α increased markedly. In contrast, levels of MaR-1 decreased significantly at this time, suggesting that MaR-1 may serve as an early-stage biomarker of sepsis. All cytokine levels were considerably higher by the sixteenth hour.

Given their modulatory effects on cytokines and potential to reduce morbidity and mortality, the administration of cetirizine and dexamethasone—either individually or in combination—may represent an effective therapeutic option in the management of sepsis.

## Figures and Tables

**Table 1:** Comparison of body temperatures (°C) in rats in the control and experimental groups before the experiment and after cetirizine and dexamethasone administration.

Experiment Group	Just Before to Start Experiment	4th Hour After Experiment Started	16th Hour After Experiment Started
G1 (n: 7)	37.14 ± 0.18	37.14 ± 0.18	37.13 ± 0.19
G2 (n: 7)	37.24 ± 0.08	36.74 ± 0.52	37.24 ± 0.25
G3 (n: 7)	37.25 ± 0.19	35.65 ± 0.62 **^a^**	38.36 ± 0.26
G4 (n: 7)	37.06 ± 0.24	36.08 ± 0.53	38.70 ± 0.23 **^b^**^,**f**^
G5 (n: 7)	36.97 ± 0.39	35.91 ± 0.76 **^c^**	38.48 ± 0.17
G6 (n: 7)	37.14 ± 0.21	35.98 ± 0.26 **^d^**	38.50 ± 0.28 **^d^**
G7 (n: 7)	37.10 ± 0.16	36.07 ± 0.25 **^e^**	38.36 ± 0.31

Body temperatures were compared within the experimental groups depending on different time periods (in vertical colons); ^a^: G1 vs. G3; ^b^: G1 vs. G4; ^c^: G1 vs. G5; ^d^: G1 vs. G6; ^e^: G1 vs. G7; ^f^: G2 vs. G4. Statistical comparisons between groups were evaluated using the Kruskal–Wallis H test (Dunn’s post hoc test). G: Group. Group 1: Control. Group 2: Sham. Group 3: Sepsis. Group 4: Sepsis + Saline. Group 5: Sepsis + Cetirizine. Group 6: Sepsis + Dexamethasone. Group 7: Sepsis + Cetirizine + Dexamethasone.

**Table 2:** Histopathological scores (mean ± SD) for hepatic inflammation, hepatocellular necrosis, vascular congestion, and Kupffer cell activation across experimental groups (G1–G7).

Parameter	G1 (n: 7)	G2 (n: 7)	G3 (n: 7)	G4 (n: 7)	G5 (n: 7)	G6 (n: 7)	G7 (n: 7)
Inflammation	1.00 ± 0.00	1.02 ± 0.01 **^a^**	2.17 ± 0.41 **^a^**	1.20 ± 0.45 **^a^**	1.29 ± 0.49 **^b^**	1.83 ± 0.75 **^b^**	1.86 ± 1.07 **^a^**
Necrosis	1.00 ± 0.00	1.00 ± 0.00 **^a^**	2.83 ± 0.75 **^a^**	2.57 ± 0.79 **^a^**	2.43 ± 0.53 **^a^**	2.33 ± 0.82 **^a^**	2.20 ± 0.84 **^a^**
Congestion	1.00 ± 0.00	1.00 ± 0.00 **^a^**	2.50 ± 0.84 **^a^**	2.43 ± 0.53 **^a^**	2.14 ± 0.69 **^a^**	1.67 ± 0.82 **^b,c,d^**	1.80 ± 0.84**^a^**
Kupffer activation	1.00 ± 0.00	1.00 ± 0.00 **^a^**	3.00 ± 0.00 **^a^**	2.14 ± 0.38 **^a,b^**	2.00 ± 0.68 **^a,b^**	2.67 ± 0.52 **^a,b^**	2.20 ± 0.45 **^a,b^**

Data are expressed as mean ± standard deviation (SD). Different superscript letters within the same row indicate statistically significant differences between groups (*p* < 0.05). Groups sharing the same letter are not significantly different from each other. ^a^: compared vs. G1; ^b^: compared vs. G3; ^c^: compared vs. G4; ^d^: compared vs. G5. G: Group. Group 1: Control. Group 2: Sham. Group 3: Sepsis. Group 4: Sepsis + Saline. Group 5: Sepsis + Cetirizine. Group 6: Sepsis + Dexamethasone. Group 7: Sepsis + Cetirizine + Dexamethasone.

**Table 3:** Comparison of the effects of cetirizine and dexamethasone on WBC, NEU, LYM, MO, PLT values in rats in the control and experimental groups at the 4th hour and 16th hour of the experiment.

Time	G	WBC (mm^3^/μL)	NEU (mm^3^/μL)	LYM (mm^3^/μL)	MO (mm^3^/μL)	PLT (mm^3^/μL)
4th hour	G1 (n: 7)	6.75 ± 1.66	4.48 ± 1.17	2.84 ± 0.92	0.49 ± 0.18	252.74 ± 62.14
	G2 (n: 7)	6.67 ± 1.11	4.36 ± 1.22	2.77 ± 0.74	0.46 ± 0.22	247.42 ± 62.11
	G3 (n: 7)	8.88 ± 1.74 **^a^**^,**b**^	5.28 ± 1.42 **^a^**^,**b**^	2.23 ± 0.86 **^a^**^,**b**^	0.54 ± 0.28	387.12 ± 76.22 **^a^**^,**b**^
	G4 (n: 7)	8.72 ± 1.88 **^a^**^,**b**^	5.32 ± 1.56 **^a^**^,**b**^	2.27 ± 0.82 **^a^**^,**b**^	0.48 ± 0.22	398.16 ± 84.46 **^a^**^,**b**^
	G5 (n: 7)	8.74 ± 1.88 **^a^**^,**b**^	5.01 ± 1.44 **^b^**	2.38 ± 0.76 **^a^**	0.46 ± 0.32	374.22 ± 68.86 **^a^**^,**b**^
	G6 (n: 7)	8.66 ± 1.44 **^a^**^,**b**^	4.98 ± 1.68 **^b^**	2.42 ± 0.98	0.42 ± 0.22	372.66 ± 72.94 **^a^**^,**b**^
	G7 (n: 7)	8.52 ± 1.68 **^b^**	4.78 ± 1.62	2.48 ± 0.66	0.47 ± 0.18	368.62 ± 83.66
16th hour	G1 (n: 7)	6.67 ± 1.34	4.40 ± 0.92	2.92 ± 0.84	0.47 ± 0.17	247.74 ± 51.09
	G2 (n: 7)	6.42 ± 1.30	4.37 ± 0.92	2.78 ± 0.62	0.45 ± 0.28	236.52 ± 48.12
	G3 (n: 7)	9.18 ± 1.92 **^a^**^,**b**^	5.78 ± 1.12 **^a^**^,**b**^	1.98 ± 0.60 **^a^**^,**b**^	0.62 ± 0.26	424.29 ± 92.38 **^a^**^,**b**^
	G4 (n: 7)	9.02 ± 2.13 **^a^**^,**b**^	5.82 ± 1.42 **^a^**^,**b**^	2.02 ± 0.68 **^a^**^,**b**^	0.59 ± 0.20	428. 02 ± 93.18 **^a^**^,**b**^
	G5 (n: 7)	8.96 ± 1.76 **^a^**^,**b**^	5.56 ± 1.72 **^a^**^,**b**^	2.52 ± 0.83	0.53 ± 0.38	402.22 ± 63.17 **^b^**
	G6 (n: 7)	8.86 ± 1.44 **^b^**	5.64 ± 1.56 **^a^**^,**b**^	2.48 ± 0.92	0.51 ± 0.26	412.21 ± 81.14 **^a^**^,**b**^
	G7 (n: 7)	8.76 ± 1.80 **^b^**	5.28 ± 1.74	2.62 ± 0.66 **^c^**^,**d**^	0.50 ± 0.19	398.18 ± 77.42 **^b^**

Vertical comparisons within the table; ^a^: compared vs. G1; ^b^: compared vs. G2; ^c^: compared vs. G3; ^d^: compared vs. G4. The *p* value of all comparisons is less than 0.05. Statistical comparisons between groups were evaluated using the Kruskal–Wallis H test (Dunn’s post hoc test). The *p* value of all comparisons is less than 0.05. WBC: White blood cell; PLT: Platelet; NEU: Neutrophil; LYM: Lymphocyte; MO: Monocyte; G: Group; Group 1: Control. Group 2: Sham. Group 3: Sepsis. Group 4: Sepsis + Saline. Group 5: Sepsis + Cetirizine. Group 6: Sepsis + Dexamethasone. Group 7: Sepsis + Cetirizine + Dexamethasone.

**Table 4:** Positive and negative relationships between variables.

Positive Related Groups	*r* Value	*p* Value	Negative Related Groups	*r* Value	*p* Value
BT (16 h)–TNF-α (4 h)	+0.836	0.000	MaR-1 (4 h)–TNF-α (4 h)	−0.904	0.000
MaR-1 (4 h)–MaR-1 (16 h)	+0.962	0.000	MaR-1 (4 h)–IL-1 (4 h)	−0.960	0.000
TNF-α (4 h)–IL-1 (4 h)	+0.931	0.000	MaR-1 (4 h)–TNF-α (16 h)	−0.891	0.000
TNF-α (4 h)–TNF-α (16 h)	+0.974	0.000	MaR-1 (4 h)–IFN-γ (16 h)	−0.929	0.000
TNF-α (4 h)–IFN-γ (16 h)	+0.914	0.000	MaR-1 (4 h)–IL-2 (16 h)	−0.953	0.000
TNF-α (4 h)–IL-1 (16 h)	+0.914	0.000	MaR-1 (4 h)–IL-6 (16 h)	−0.923	0.000
TNF-α (4 h)–IL-2 (16 h)	+0.890	0.000	MaR-1 (4 h)–IL-8 (16 h)	−0.923	0.000
TNF-α (4 h)–IL-6 (16 h)	+0.892	0.000	MaR-1 (4 h)–IL-10 (16 h)	−0.935	0.000
TNF-α (4 h)–IL-8 (16 h)	+0.937	0.000	TNF-α (4 h)–MaR-1 (16 h)	−0.913	0.000
TNF-α (4 h)–IL-10 (16 h)	+0.901	0.000	IL-1 (4 h)–MaR-1 (16 h)	−0.948	0.000
IL-1 (4 h)–TNF-α (16 h)	+0.917	0.000	MaR-1 (16 h)–TNF-α (16 h)	−0.882	0.000
IL-1 (4 h)–IFN-γ (16 h)	+0.949	0.000	MaR-1 (16 h)–IFN-γ (16 h)	−0.924	0.000
IL-1 (4 h)–IL-1 (16 h)	+0.992	0.000	MaR-1 (16 h)–IL-1 (16 h)	−0.940	0.000
IL-1 (4 h)–IL-2 (16 h)	+0.934	0.000	MaR-1 (16 h)–IL-2 (16 h)	−0.917	0.000
IL-1 (4 h)–IL-6 (16 h)	+0.938	0.000	MaR-1 (16 h)–IL-6 (16 h)	−0.891	0.000
IL-1 (4 h)–IL-8 (16 h)	+0.937	0.000	MaR-1 (16 h)–IL-8 (16 h)	−0.881	0.000
IL-1 (4 h)–IL-10 (16. h)	+0.923	0.000	MaR-1 (16 h)–IL-10 (16 h)	−0.920	0.000
TNF-α (16 h)–IFN-γ (16 h)	+0.862	0.000			
TNF-α (16 h)–IL-1 (16 h)	+0.892	0.000			
TNF-α (16 h)–IL-2 (16 h)	+0.871	0.000			
TNF-α (16 h)–IL-6 (16 h)	+0.864	0.000			
TNF-α (16 h)–IL-8 (16 h)	+0.916	0.000			
TNF-α (16 h)–IL-10 (16 h)	+0.959	0.000			
IFN-γ (16 h)–IL-1 (16 h)	+0.946	0.000			
IFN-γ (16 h)–IL-2 (16 h)	+0.915	0.000			
IFN-γ (16 h)–IL-8 (16 h)	+0.884	0.000			
IFN-γ (16 h)–IL-10 (16 h)	+0.959	0.000			
IL-1 (16 h)–IL-2 (16 h)	+0.904	0.000			
IL-1 (16 h)–IL-6 (16 h)	+0.947	0.000			
IL-1 (16 h)–IL-8 (16 h)	+0.927	0.000			
IL-1 (16 h)–IL-10 (16 h)	+0.951	0.000			
IL-2 (16 h)–IL-6 (16 h)	+0.902	0.000			
IL-2 (16 h)–IL-8 (16 h)	+0.902	0.000			
IL-2 (16 h)–IL-10 (16 h)	+0.886	0.000			
IL-6 (16 h)–IL-8 (16 h)	+0.900	0.000			
IL-6 (16 h)–IL-10 (16 h)	+0.929	0.000			
IL-8 (16 h)–IL-10 (16 h)	+0.881	0.000			

BT: Body temperature. h: Hour. IFN-γ: Interferon gamma. IL: Interleukin. MaR-1: Maresin-1. TNF-α: Tumor necrosis factor-α. Statistical correlations test between groups were evaluated using the Spearman correlation test.

## Data Availability

The datasets generated and/or analyzed during this study are available from the corresponding author upon reasonable request.

## Abbreviations

CLP	Cecal ligation and puncture
CV	Coefficients of variation
DHA	Docosahexaenoic acid
FÜHADYEK	Fırat University Local Ethics Committee Animal Experiments
LPS	Lipopolysaccharides
LYM	Lymphocyte
MaR-1	Maresin-1
MCP	Monocyte chemoattractant protein
MO	Monocyte
NEU	Neutrophil
NK	Natural killer
PLT	Platelet
TNF-α	Tumor necrosis factor-alpha
WBC	White blood cell

## References

[1] Dolin H.H., Papadimos T.J., Chen X., Pan Z.K. (2019). Characterization of Pathogenic Sepsis Etiologies and Patient Profiles: A Novel Approach to Triage and Treatment. Microbiol. Insights.

[2] Hotchkiss R.S., Moldawer L.L., Opal S.M., Reinhart K., Turnbull I.R., Vincent J.L. (2016). Sepsis and septic shock. Nat. Rev. Dis. Primers.

[3] Chousterman B.G., Swirski F.K., Weber G.F. (2017). Cytokine storm and sepsis disease pathogenesis. Semin. Immunopathol..

[4] Vourc’h M., Roquilly A., Broquet A., David G., Hulin P., Jacqueline C., Caillon J., Retiere C., Asehnoune K. (2017). Exoenzyme T Plays a Pivotal Role in the IFN-γ Production after Pseudomonas Challenge in IL-12 Primed Natural Killer Cells. Front. Immunol..

[5] McMasters M., Mora J. (2025). Addressing Meta-Inflammation in the Comprehensive Management of Chronic Pain. Cureus.

[6] Bouchery T., Harris N. (2019). Neutrophil-macrophage cooperation and its impact on tissue repair. Immunol. Cell Biol..

[7] Liu W.C., Yang Y.H., Wang Y.C., Chang W.M., Wang C.W. (2023). Maresin: Macrophage Mediator for Resolving Inflammation and Bridging Tissue Regeneration—A System-Based Preclinical Systematic Review. Int. J. Mol. Sci..

[8] Saito-Sasaki N., Sawada Y., Nakamura M. (2022). Maresin-1 and Inflammatory Disease. Int. J. Mol. Sci..

[9] Li D., Wang M., Ye J., Zhang J., Xu Y., Wang Z., Zhao M., Ye D., Wan J. (2021). Maresin 1 alleviates the inflammatory response, reduces oxidative stress and protects against cardiac injury in LPS-induced mice. Life Sci..

[10] Kourtzelis I., Hajishengallis G., Chavakis T. (2020). Phagocytosis of Apoptotic Cells in Resolution of Inflammation. Front. Immunol..

[11] Canonica G.W., Blaiss M. (2011). Antihistaminic, anti-inflammatory, and antiallergic properties of the nonsedating second-generation antihistamine desloratadine: A review of the evidence. World Allergy Organ. J..

[12] Kumar A., Husain N., Anbazhagan A.N., Jayawardena D., Priyamvada S., Singhal M., Jain C., Kaur P., Guzman G., Saksena S. (2025). Dexamethasone Upregulates the Expression of the Human SLC26A3 (DRA, Down-Regulated in Adenoma) Transporter (an IBD Susceptibility Gene) in Intestinal Epithelial Cells and Attenuates Gut Inflammation. Inflamm. Bowel Dis..

[13] Zappia C.D., Torralba-Agu V., Echeverria E., Fitzsimons C.P., Fernández N., Monczor F. (2021). Antihistamines Potentiate Dexamethasone Anti-Inflammatory Effects. Impact on Glucocorticoid Receptor-Mediated Expression of Inflammation-Related Genes. Cells.

[14] Hattori M., Yamazaki M., Ohashi W., Tanaka S., Hattori K., Todoroki K., Fujimori T., Ohtsu H., Matsuda N., Hattori Y. (2016). Critical role of endogenous histamine in promoting end-organ tissue injury in sepsis. Intensive Care Med. Exp..

[15] Pitre T., Drover K., Chaudhuri D., Zeraaktkar D., Menon K., Gershengorn H.B., Jayaprakash N., Spencer-Segal J.L., Pastores S.M., Nei A.M. (2024). Corticosteroids in Sepsis and Septic Shock: A Systematic Review, Pairwise, and Dose-Response Meta-Analysis. Crit. Care Explor..

[16] Duffy B.A., Chun K.P., Ma D., Lythgoe M.F., Scott R.C. (2014). Dexamethasone exacerbates cerebral edema and brain injury following lithium-pilocarpine induced status epilepticus. Neurobiol. Dis..

[17] Waage A. (1987). Production and clearance of tumor necrosis factor in rats exposed to endotoxin and dexamethasone. Clin. Immunol. Immunopathol..

[18] Sun S., Wang L., Wang J., Chen R., Pei S., Yao S., Lin Y., Yao C., Xia H. (2023). Maresin1 prevents sepsis-induced acute liver injury by suppressing NF-κB/Stat3/MAPK pathways, mitigating inflammation. Heliyon.

[19] Mahmoud J., Bovy M.A., Heming N., Annane D. (2025). Corticosteroids in sepsis. J. Intensive Med..

[20] Lowry P., Blanco T., Santiago-Delpín E.A. (1977). Histamine and sympathetic blockade in septic shock. Am. Surg..

[21] Firzli T.R., Sathappan S., Antwi-Amoabeng D., Beutler B.D., Ulanja M.B., Madhani-Lovely F. (2023). Association between histamine 2 receptor antagonists and sepsis outcomes in ICU patients: A retrospective analysis using the MIMI-IV database. J. Anesth. Analg. Crit. Care.

[22] Aydin Y. (2023). Deneysel sepsis oluşturulan ratlarda deksametazon ve setirizin’in TNF-α, İFN-γ İnterlökin (IL)-1β, IL-2, IL-6, IL-8, IL-10 ve Maresin 1 düzeylerine etkileri. Ph.D. Thesis.

[23] Wichterman K.A., Baue A.E., Chaudry I.H. (1980). Sepsis and septic shock—A review of laboratory models and a proposal. J. Surg. Res..

[24] Ionov I.D., Gorev N.P., Roslavtseva L.A., Frenkel D.D. (2018). Cetirizine and thalidomide synergistically inhibit mammary tumorigenesis and angiogenesis in 7,12-dimethylbenz(a)anthracene-treated rats. Anticancer Drugs.

[25] Battiston F.G., Dos Santos C., Barbosa A.M., Sehnem S., Leonel E.C.R., Taboga S.R., Anselmo-Franci J.A., Lima F.B., Rafacho A. (2017). Glucose homeostasis in rats treated with 4-vinylcyclohexene diepoxide is not worsened by dexamethasone treatment. J. Steroid Biochem. Mol. Biol..

[26] Aydin S., Emre E., Ugur K., Aydin M.A., Sahin İ., Cinar V., Akbulut T. (2025). An overview of ELISA: A review and update on best laboratory practices for quantifying peptides and proteins in biological fluids. J. Int. Med. Res..

[27] Aydin S. (2015). A short history, principles, and types of ELISA, and our laboratory experience with peptide/protein analyses using ELISA. Peptides.

[28] Dellinger R.P., Levy M.M., Rhodes A., Annane D., Gerlach H., Opal S.M., Sevransky J.E., Sprung C.L., Douglas I.S., Jaeschke R. (2013). Surviving Sepsis Campaign: International Guidelines for Management of Severe Sepsis and Septic Shock, 2012. Intensive Care Med..

[29] Güler M.C., Tanyeli A., Eraslan E., Çomakli S., Bayir Y. (2022). Cecal Ligation and Puncture-Induced Sepsis Model in Rats. Lab. Hayv. Bil. Uyg. Derg..

[30] Prescott H.C., Angus D.C. (2018). Enhancing Recovery from Sepsis: A Review. JAMA.

[31] Freise H., Brückner U.B., Spiegel H.U. (2001). Animal models of sepsis. J. Investig. Surg..

[32] Remick D.G., Newcomb D.E., Bolgos G.L., Call D.R. (2000). Comparison of the mortality and inflammatory response of two models of sepsis: Lipopolysaccharide vs. cecal ligation and puncture. Shock.

[33] Díaz M., Becker D.E. (2010). Thermoregulation: Physiological and clinical considerations during sedation and general anesthesia. Anesth. Prog..

[34] Léon K., Pichavant-Rafini K., Ollivier H., Monbet V., L’Her E. (2015). Does induction time of mild hypothermia influence the survival duration of septic rats?. Ther. Hypothermia Temp. Manag..

[35] Zhang Y., Feng Y., Chen F., Yu J., Liu X., Liu Y., Ouyang J., Liang M., Zhu Y., Zou L. (2023). Insight into the mechanisms of therapeutic hypothermia for asphyxia cardiac arrest using a comprehensive approach of GC-MS/MS and UPLC-Q-TOF-MS/MS based on serum metabolomics. Heliyon.

[36] Heming N., Sivanandamoorthy S., Meng P., Bounab R., Annane D. (2018). Immune Effects of Corticosteroids in Sepsis. Front. Immunol..

[37] Ruiz S., Vardon-Bounes F., Merlet-Dupuy V., Conil J.M., Buléon M., Fourcade O., Tack I., Minville V. (2016). Sepsis modeling in mice: Ligation length is a major severity factor in cecal ligation and puncture. Intensive Care Med. Exp..

[38] Song R., Kim J., Yu D., Park C., Park J. (2012). Kinetics of IL-6 and TNF-α changes in a canine model of sepsis induced by endotoxin. Veter. Immunol. Immunopathol..

[39] Ito A., Sawamoto A., Okuyama S., Nakajima M. (2025). Second-Generation H_1_-Receptor Antagonists, Mequitazine, Azelastine and Desloratadine Activate Caspase-8, Caspase-3, and Gasdermin E and Induce Cell Death Showing Pyroptotic-Like Features in Macrophages at Excessively High Concentrations. Biol. Pharm. Bull..

[40] Abraham S.M., Lawrence T., Kleiman A., Warden P., Medahalli M., Tuckermann J., Saklatvala J., Clark A.R. (2006). Antiinflammatory effects of dexamethasone are partly dependent on induction of dual specificity phosphatase 1. J. Exp. Med..

[41] de Almeida A.R., Dantas A.T., Pereira M.C., Cordeiro M.F., Gonçalves R.S.G., de Melo Rêgo M.J.B., da Rocha Pitta I., Duarte A.L.B.P., da Rocha Pitta M.G. (2019). Dexamethasone inhibits cytokine production in PBMC from systemic sclerosis patients. Inflammopharmacology.

[42] Smits H.H., Grünberg K., Derijk R.H., Sterk P.J., Hiemstra P.S. (1998). Cytokine release and its modulation by dexamethasone in whole blood following exercise. Clin. Exp. Immunol..

[43] Fang H., Liu A., Chen X., Cheng W., Dirsch O., Dahmen U. (2018). The severity of LPS induced inflammatory injury is negatively associated with the functional liver mass after LPS injection in rat model. J. Inflamm..

[44] Hao Y., Zheng H., Wang R.H., Li H., Yang L.L., Bhandari S., Liu Y.J., Han J., Smith F.G., Gao H.C. (2019). Maresin1 Alleviates Metabolic Dysfunction in Septic Mice: A 1H NMR-Based Metabolomics Analysis. Mediat. Inflamm..

[45] Li R., Wang Y., Ma Z., Ma M., Wang D., Xie G., Yin Y., Zhang P., Tao K. (2016). Maresin 1 Mitigates Inflammatory Response and Protects Mice from Sepsis. Mediat. Inflamm..

[46] Page M.J., Kell D.B., Pretorius E. (2022). The Role of Lipopolysaccharide-Induced Cell Signalling in Chronic Inflammation. Chronic Stress.

[47] Sun S., Wang J., Wang J., Wang F.Q., Yao S.L., Xia H.F. (2019). Maresin 1 Mitigates Sepsis-Associated Acute Kidney Injury in Mice via Inhibition of the NF-κB/STAT3/MAPK Pathways. Front. Pharmacol..

[48] Alexander J.J., Jacob A., Cunningham P., Hensley L., Quigg R.J. (2008). TNF is a key mediator of septic encephalopathy acting through its receptor, TNF receptor-1. Neurochem. Int..

[49] Hubbi S., Hao S., Epps J., Ferreri N.R. (2025). Tumour necrosis factor-alpha at the intersection of renal epithelial and immune cell function. J. Physiol..

[50] Akgül Y., Akgül Ö., Kozat S., Özkan C., Kaya A., Yılmaz N. (2019). Evaluation of intercellular adhesion molecule-1 (ICAM-1), Tumor necrosis factor α (TNF-α), Interleukins (IL-6, IL-8) and C-reactive protein (CRP) levels in neonatal calves with presumed septicemia. Van Veter. J..

[51] Shen X.F., Cao K., Jiang J.P., Guan W.X., Du J.F. (2017). Neutrophil dysregulation during sepsis: An overview and update. J. Cell Mol. Med..

[52] León I.C., Quesada-Vázquez S., Sáinz N., Guruceaga E., Escoté X., Moreno-Aliaga M.J. (2020). Effects of Maresin 1 (MaR1) on Colonic Inflammation and Gut Dysbiosis in Diet-Induced Obese Mice. Microorganisms.

[53] Rybacka-Chabros B., Pietrzak A., Milanowski J., Chabros P. (2014). Influence of prednisone on serum level of tumor necrosis factor alpha, interferon gamma and interleukin-1 beta, in active pulmonary tuberculosis. Pol. J. Public Health.

[54] Shi Z., Fultz R.S., Engevik M.A., Gao C., Hall A., Major A., Mori-Akiyama Y., Versalovic J. (2019). Distinct roles of histamine H1- and H2-receptor signaling pathways in inflammation-associated colonic tumorigenesis. Am. J. Physiol. Gastrointest. Liver Physiol..

[55] Shin D.I., Banning U., Kim Y.M., Verheyen J., Hannen M., Bönig H., Körholz D. (1999). Interleukin 10 inhibits TNF-alpha production in human monocytes independently of interleukin 12 and interleukin 1 beta. Immunol. Investig..

[56] Mertowska P., Mertowski S., Smarz-Widelska I., Grywalska E. (2022). Biological Role, Mechanism of Action and the Importance of Interleukins in Kidney Diseases. Int. J. Mol. Sci..

[57] Nie J., Zhou L., Tian W., Liu X., Yang L., Yang X., Zhang Y., Wei S., Wang D.W., Wei J. (2025). Deep insight into cytokine storm: From pathogenesis to treatment. Signal Transduct. Target. Ther..

[58] Okusawa S., Gelfand J.A., Ikejima T., Connolly R.J., Dinarello C.A. (1988). Interleukin 1 induces a shock-like state in rabbits. Synergism with tumor necrosis factor and the effect of cyclooxygenase inhibition. J. Clin. Investig..

[59] Zhao W., Jia L., Yang H.J., Xue X., Xu W.X., Cai J.Q., Guo R.J., Cao C.C. (2018). Taurine enhances the protective effect of Dexmedetomidine on sepsisinduced acute lung injury via balancing the immunological system. Biomed. Pharmacother..

[60] Khan S., Malladi V.S., von Itzstein M.S., Mu-Mosley H., Fattah F.J., Liu Y., Gwin M.E., Park J.Y., Cole S.M., Bhalla S. (2025). Innate and adaptive immune features associated with immune-related adverse events. J. Immunother. Cancer.

[61] Meldrum D.R., Ayala A., Perrin M.M., Ertel W., Chaudry I.H. (1991). Diltiazem restores IL-2, IL-3, IL-6, and IFN-gamma synthesis and decreases host susceptibility to sepsis following hemorrhage. J. Surg. Res..

[62] Qiu G., Wang C., Smith R., Harrison K., Yin K. (2001). Role of IFN-gamma in bacterial containment in a model of intra-abdominal sepsis. Shock.

[63] Gharamti A., Samara O., Monzon A., Scherger S., DeSanto K., Sillau S., Franco-Paredes C., Henao-Martínez A., Shapiro L. (2021). Association between cytokine levels, sepsis severity and clinical outcomes in sepsis: A quantitative systematic review protocol. BMJ Open.

[64] Kim E.Y., Ner-Gaon H., Varon J., Cullen A.M., Guo J., Choi J., Barragan-Bradford D., Higuera A., Pinilla-Vera M., Short S.A. (2020). Post-sepsis immunosuppression depends on NKT cell regulation of mTOR/IFN-γ in NK cells. J. Clin. Investig..

[65] Li Q., Wu C., Liu Z., Zhang H., Du Y., Liu Y., Song K., Shi Q., Li R. (2019). Increased TLR4 Expression Aggravates Sepsis by Promoting IFN-γ Expression in CD38^−/−^ Mice. J. Immunol. Res..

[66] Romero C.R., Herzig D.S., Etogo A., Nunez J., Mahmoudizad R., Fang G., Murphey E.D., Toliver-Kinsky T., Sherwood E.R. (2010). The role of interferon-γ in the pathogenesis of acute intra-abdominal sepsis. J. Leukoc. Biol..

[67] Gigante M., Ranieri E. (2014). In vitro\ex vivo generation of cytotoxic T lymphocytes. Methods Mol. Biol..

[68] Leonard W.J., Depper J.M., Crabtree G.R., Rudikoff S., Pumphrey J., Robb R.J., Krönke M., Svetlik P.B., Peffer N.J., Waldmann T.A. (1984). Molecular cloning and expression of cDNAs for the human interleukin-2 receptor. Nature.

[69] Bachmann M.F., Kopf M. (2002). Balancing protective immunity and immunopathology. Curr. Opin. Immunol..

[70] Zhang Y., Kong Q., Fan J., Zhao H. (2025). Interleukin-2 and its receptors: Implications and therapeutic prospects in immune-mediated disorders of central nervous system. Pharmacol. Res..

[71] Endo S., Inada K., Inoue Y. (1992). Two types of septic shock classified by the plasma levels of cytokines and endotoxin. Circ. Shock.

[72] Ma L., Zhang H., Yin Y.L., Guo W.Z., Ma Y.Q., Wang Y.B., Shu C., Dong L.Q. (2016). Role of interleukin-6 to differentiate sepsis from non-infectious systemic inflammatory response syndrome. Cytokine.

[73] Song J., Park D.W., Moon S., Cho H.J., Park J.H., Seok H., Choi W.S. (2019). Diagnostic and prognostic value of interleukin-6, pentraxin 3, and procalcitonin levels among sepsis and septic shock patients: A prospective controlled study according to the Sepsis-3 definitions. BMC Infect. Dis..

[74] Spittler A., Razenberger M., Kupper H., Kaul M., Hackl W., Boltz-Nitulescu G., Függer R., Roth E. (2000). Relationship between interleukin-6 plasma concentration in patients with sepsis, monocyte phenotype, monocyte phagocytic properties, and cytokine production. Clin. Infect. Dis..

[75] Schüttler J., Neumann S. (2015). Interleukin-6 as a prognostic marker in dogs in an intensive care unit. Veter. Clin. Pathol..

[76] Badea I.M., Azamfirei R., Grigorescu B.L., Ráduly G., Hutanu A., Petrişor M., Lazăr A.E., Almásy E., Fodor R.Ş., Man A. (2019). The role of interleukin-6 as an early predictor of sepsis in a murine sepsis model. Rom. J. Morphol. Embryol..

[77] Cong S., Ma T., Di X., Tian C., Zhao M., Wang K. (2021). Diagnostic value of neutrophil CD64, procalcitonin, and interleukin-6 in sepsis: A meta-analysis. BMC Infect. Dis..

[78] Weiss S.L., Fitzgerald J.C., Laskin B.L., Singh R., Artis A.S., Vohra A., Tsemberis E., Kierian E., Lau K.C., Campos A.B. (2025). Time Course of Kidney Injury Biomarkers in Children with Septic Shock: Nested Cohort Study Within the Pragmatic Pediatric Trial of Balanced Versus Normal Saline Fluid in Sepsis Trial. Pediatr. Crit. Care Med..

[79] Harbarth S., Holeckova K., Froidevaux C., Pittet D., Ricou B., Grau G.E., Vadas L., Pugin J., Geneva Sepsis Network (2001). Diagnostic value of procalcitonin, interleukin-6, and interleukin-8 in critically ill patients admitted with suspected sepsis. Am. J. Respir. Crit. Care Med..

[80] Yang J., Yang L., Wang Y., Huai L., Shi B., Zhang D., Xu W., Cui D. (2025). Interleukin-6 related signaling pathways as the intersection between chronic diseases and sepsis. Mol. Med..

[81] Hack C.E., Hart M., van Schijndel R.J., Eerenberg A.J., Nuijens J.H., Thijs L.G., Aarden L.A. (1992). Interleukin-8 in sepsis: Relation to shock and inflammatory mediators. Infect. Immun..

[82] Martins E.C., da Fe Silveira L., Viegas K., Beck A.D., Júnior G.F., Cremonese R.V., Lora P.S. (2019). Neutrophil-lymphocyte ratio in the early diagnosis of sepsis in an intensive care unit: A case-control study. Rev. Bras. Ter. Intensiv..

[83] Naess A., Nilssen S.S., Mo R., Eide G.E., Sjursen H. (2017). Role of neutrophil to lymphocyte and monocyte to lymphocyte ratios in the diagnosis of bacterial infection in patients with fever. Infection.

[84] Jung E., Romero R., Suksai M., Gotsch F., Chaemsaithong P., Erez O., Conde-Agudelo A., Gomez-Lopez N., Berry S.M., Meyyazhagan A. (2024). Clinical chorioamnionitis at term: Definition, pathogenesis, microbiology, diagnosis, and treatment. Am. J. Obstet. Gynecol..

[85] Kraft R., Herndon D.N., Finnerty C.C., Cox R.A., Song J., Jeschke M.G. (2015). Predictive Value of IL-8 for Sepsis and Severe Infections After Burn Injury: A Clinical Study. Shock.

[86] Oberholzer A., Oberholzer C., Moldawer L.L. (2002). Interleukin-10: A complex role in the pathogenesis of sepsis syndromes and its potential as an anti-inflammatory drug. Crit. Care Med..

[87] Hu J., Tang Z., Xu J., Ge W., Hu Q., He F., Zheng G., Jiang L., Yang Z., Tang W. (2019). The inhibitor of interleukin-3 receptor protects against sepsis in a rat model of cecal ligation and puncture. Mol. Immunol..

[88] Calzavacca P., Lankadeva Y.R., Bailey S.R., Bailey M., Bellomo R., May C.N. (2014). Effects of selective β1-adrenoceptor blockade on cardiovascular and renal function and circulating cytokines in ovine hyperdynamic sepsis. Crit. Care.

[89] Rutai A., Zsikai B., Tallósy S.P., Érces D., Bizánc L., Juhász L., Poles M.Z., Sóki J., Baaity Z., Fejes R. (2022). A Porcine Sepsis Model with Numerical Scoring for Early Prediction of Severity. Front. Med..

[90] Senousy S.R., Ahmed A.F., Abdelhafeez D.A., Khalifa M.M.A., Abourehab M.A.S., El-Daly M. (2022). Alpha-Chymotrypsin Protects Against Acute Lung, Kidney, and Liver Injuries and Increases Survival in CLP-Induced Sepsis in Rats Through Inhibition of TLR4/NF-κB Pathway. Drug Des. Devel. Ther..

[91] Gabbia D., Pozzo L., Zigiotto G., Roverso M., Sacchi D., Pozza A.D., Carrara M., Bogialli S., Floreani A., Guido M. (2018). Dexamethasone counteracts hepatic inflammation and oxidative stress in cholestatic rats via CAR activation. PLoS ONE.

[92] Sadowska-Woda I., Bieszczad-Bedrejczuk E., Rachel M. (2010). Influence of desloratadine on selected oxidative stress markers in patients between 3 and 10 years of age with allergic perennial rhinitis. Eur. J. Pharmacol..

